# Complex Interactions between Soil-Transmitted Helminths and Malaria in Pregnant Women on the Thai-Burmese Border

**DOI:** 10.1371/journal.pntd.0000887

**Published:** 2010-11-16

**Authors:** Machteld Boel, Verena I. Carrara, Marcus Rijken, Stephane Proux, Mathieu Nacher, Mupawjay Pimanpanarak, Moo Koo Paw, Oh Moo, Hser Gay, Wendi Bailey, Pratap Singhasivanon, Nicholas J. White, François Nosten, Rose McGready

**Affiliations:** 1 Shoklo Malaria Research Unit, Tak, Thailand; 2 Academic Medical Center, Amsterdam, The Netherlands; 3 Department Obstetrics, University Medical Center, Utrecht, The Netherlands; 4 Centre d'Investigation Clinique Epidemiologie Clinique Antilles Guyane CIC EC CIE 802, Cayenne, French Guiana; 5 Liverpool School of Tropical Medicine, Liverpool, United Kingdom; 6 Faculty of Tropical Medicine, Mahidol University, Bangkok, Thailand; 7 Centre for Tropical Medicine, Nuffield Department of Clinical Medicine, University of Oxford, Oxford, United Kingdom; London School of Hygiene & Tropical Medicine, United Kingdom

## Abstract

**Background:**

Deworming is recommended by the WHO in girls and pregnant and lactating women to reduce anaemia in areas where hookworm and anaemia are common. There is conflicting evidence on the harm and the benefits of intestinal geohelminth infections on the incidence and severity of malaria, and consequently on the risks and benefits of deworming in malaria affected populations. We examined the association between geohelminths and malaria in pregnancy on the Thai-Burmese border.

**Methodology:**

Routine antenatal care (ANC) included active detection of malaria (weekly blood smear) and anaemia (second weekly haematocrit) and systematic reporting of birth outcomes. In 1996 stool samples were collected in cross sectional surveys from women attending the ANCs. This was repeated in 2007 when malaria incidence had reduced considerably. The relationship between geohelminth infection and the progress and outcome of pregnancy was assessed.

**Principal Findings:**

Stool sample examination (339 in 1996, 490 in 2007) detected a high prevalence of geohelminths 70% (578/829), including hookworm (42.8% (355)), *A. lumbricoides* (34.4% (285)) and *T.trichuria* (31.4% (250)) alone or in combination. A lower proportion of women (829) had mild (21.8% (181)) or severe (0.2% (2)) anaemia, or malaria 22.4% (186) (*P.vivax* monoinfection 53.3% (101/186)). *A. lumbricoides* infection was associated with a significantly decreased risk of malaria (any species) (AOR: 0.43, 95% CI: 0.23–0.84) and *P.vivax* malaria (AOR: 0.29, 95% CI: 0.11–0.79) whereas hookworm infection was associated with an increased risk of malaria (any species) (AOR: 1.66, 95% CI: 1.06–2.60) and anaemia (AOR: 2.41, 95% CI: 1.18–4.93). Hookworm was also associated with low birth weight (AOR: 1.81, 95% CI: 1.02–3.23).

**Conclusion/Significance:**

*A. lumbricoides* and hookworm appear to have contrary associations with malaria in pregnancy.

## Author Summary

Intestinal worms, particularly hookworm and whipworm, can cause anaemia, which is harmful for pregnant women. The WHO recommends deworming in pregnancy in areas where hookworm infections are frequent. Some studies indicate that coinfection with worms and malaria adversely affects pregnancy whereas other studies have shown that coinfection with worms might reduce the severity of malaria. On the Thai-Burmese border malaria in pregnancy has been an important cause of maternal death. We examined the relationship between intestinal helminth infections in pregnant women and their malaria risk in our antenatal care units. In total 70% of pregnant women had worm infections, mostly hookworm, but also roundworm and whipworm; hookworm was associated with mild anaemia although ova counts were not high. Women infected with hookworm had more malaria and their babies had a lower birth weight than women without hookworm. In contrast women with roundworm infections had the lowest rates of malaria in pregnancy. Deworming eliminates all worms. In this area it is unclear whether mass deworming would be beneficial.

## Introduction

In 1994 and 2002 the World Health Organization (WHO) recommended anthelminthics be given to girls, pregnant and lactating women to reduce the burden of anaemia in areas where hookworm and anaemia are common [1]–[3]. Published evidence suggests that mebendazole [4]–[8] or albendazole [9]–[14] administered after the first trimester of pregnancy are safe. However the advantage of routine deworming of pregnant women is debatable, with different studies presenting different results. Several studies reported that systematic anthelminthic administration was associated with less anaemia [2], [4], [6], [10], [11], [13] and with a beneficial effect on birth outcomes, reducing the rates of low birth weight [5], [7]–[9], [12], very low birth weight [15], stillbirth and perinatal death [7]. However a Cochrane review, including three prospective randomised controlled trials studying the effect of deworming in pregnancy, concluded that the evidence to date is insufficient to recommend use of antihelminthics for pregnant women after the first trimester of pregnancy [16]. A recent randomised controlled trial in Uganda showed no benefit of anthelminthic treatment on maternal anaemia, low birthweight and perinatal mortality [17].

There are also conflicting and often confusing results regarding the impact of geohelminth infections on other infectious diseases, and in particular malaria [18]–[24].While some studies have failed to find any relationship between geohelminth infection and malaria [25], others have shown an increased incidence of *P. falciparum* malaria in presence of geohelminths [26]–[28]. *Ascaris (A.) lumbricoides* infections were linked to severe *P. falciparum* malaria in Senegal [29] but they have more often been associated with a beneficial effect on malaria [20], [30]–[34]. Several immunological hypotheses, including modulation of T-helper or dendritic cell responses and cytokine induction, have been proposed to explain these interactions [35]–[40]. There have also been haematological and entomological hypotheses to explain increased incidence [19].

Data from studies specific to pregnancy and helminths are also conflicting. Hookworm, not *P. falciparum* malaria, was considered the main cause of anaemia in some [41], [42], while others reported an opposite result [43], [44] or did not find any association [15]. Maternal co-infection with *P. falciparum* and helminths resulted in a significantly lower mean birth weight than with *P. falciparum* infection alone in Nigeria and Ghana [45], [46]. Two recent studies report an association with lower rates of *P.falciparum* infection in women co-infected with *A. lumbricoides* [47], [48]. Yatich and colleagues report a 4.8 (95% 3.4–40) fold increased risk of *P.falciparum* with any geohelminth and the risk remained significant for hookworm and *A. lumbricoides* alone [49].

Two cross-sectional helminth surveys (1996 and 2007) conducted among women attending antenatal clinics on the Thai-Burmese border were reviewed to determine whether there was any association between geohelminth infection and malaria in this area, endemic for both *P. falciparum* and *P.vivax* malaria species and where there has been no systematic deworming during pregnancy.

## Methods

### Population and antenatal care

The Shoklo Malaria Research Unit (SMRU) has five established clinics on the Thai-Burmese border. One is based in the largest of the refugee camps, Maela (circa 45,000 people); the others are stretched along 100 km of the border and serve a migrant population of circa 50,000 people. Antenatal clinics (ANC) have been operational since 1986 in the camp and 1998 in the migrant population. Malaria transmission is low and seasonal [50]. Treatment is complicated by a high level of multi-drug resistant strains of *P. falciparum* [51]. There is currently no safe and effective *P. falciparum* antimalarial drug that can be offered as intermittent presumptive therapy (IPT) or prophylaxis to pregnant women. Active weekly detection and early treatment of malaria has so far been the best method to prevent maternal death from malaria in this area [52].

The ANC performs a weekly malaria smear for all women, 2^nd^ weekly haematocrit, provides routine iron and folate supplementation, and all necessary medical and obstetric care. A mother-to-child HIV transmission prevention program started in 2001 in the refugee camp and was introduced to the migrant population in 2008. HIV prevalence is low (<1.0%) and test uptake high (>90%) [53].

The antimalarial drug regimen for the treatment of *P. falciparum* in pregnant women was quinine or mefloquine mono-therapy in 1996, and quinine or artesunate with or without clindamycin in 2007. *P.vivax* episodes are treated with chloroquine alone. In 1996 women with non-severe (mild) anaemia (haematocrit between 20% and 29.9%, HB between 6.7 g/L and 10 g/L) were treated with ferrous sulphate (200 mg three times daily) and folic acid (5 mg daily) until delivery. Women with severe symptomatic anaemia (haematocrit <20%, HB <6.7 g/L) were transfused. In 2007 all women received ferrous sulphate, 200 mg daily and folic acid, 5 mg weekly, from first consultation to delivery and treatment doses as stated previously if they became anaemic. Thailand has no deworming policy for pregnant women nor do the agencies working in the refugee camps.

Women were encouraged to deliver with trained midwives in the SMRU delivery rooms, those requiring Caesarean section were transferred to the nearest Thai Hospital. Gestational age was estimated by Dubowitz score [54] in 1996 and by ultrasound (or Dubowitz for late scans) in 2007 [55]. Birth weight was measured on electronic Seca scales (accuracy 10 grams) or Salter hanging scales (accuracy 50 grams).

### Socioeconomic status

Women participating in the surveys were of similarly deprived socioeconomic groups. Refugees in the camps receive food assistance and have access to medical care, but cannot work. Migrants work hard for low wages and lack access to medical care. Both groups are poor and economically weak [56]. Housing of refugee and migrant women are the same. Houses are elevated on poles of wood and walls and floors are made of bamboo with leaf roofing. Most families have their own toilets. Flip flops (flat sandal) are normally worn on the feet in all age groups. Contact with soil that is reportedly highly contaminated with helminth is inevitable [57], more common in the rainy season and in those involved in agricultural work.

### Definitions

Miscarriage was loss of products of conception or foetus before 28 completed weeks of gestation; stillbirth was delivery of a dead foetus aged 28 weeks or more; low birth weight (LBW) was a birth weight of <2500 grams measured in the first 5 days of life, and prematurity a delivery before 37.0 weeks of gestation; congenital abnormality was considered if a major defect was present at birth.

### Stool survey procedures

The surveys in 1996 and 2007 were both conducted during the rainy season period (May–Oct) in order to allow comparison without having to take into account seasonality and because malaria peaks at that time of the year. Both surveys were exhaustive. The first survey, when SMRU only worked in the refugee camp, was to determine if worm infection was associated with anaemia. Every woman was asked to participate.

The 2nd survey was done as a response to the preparation of a border wide medical guideline. The refugee camps on the border fall under the care of different NGOs (Non-Government Organisations) and there was debate on deworming in pregnancy. Since the last survey was old it was decided to resurvey pregnant women to determine if there was a need for deworming in pregnancy. At the time of this survey SMRU also provided antenatal care for migrants who have less access to health care than refugees. Hence refugee and migrant women were surveyed if they voluntarily gave a stool sample. Every 5th woman was asked to participate.

Before each survey a general announcement was made to all pregnant women attending the ANC. Participation was voluntary. It was explained that if their stools were found positive for worms they would receive anthelminthic treatment. The importance of providing a fresh stool sample was explained.

Stool samples were examined on site. As Necator americanus and Ankylostoma duodenale ova cannot be differentiated by microscopy, the term hookworm was used. Women with a positive stool test result for hookworm, *A. lumbricoides* (roundworm) or *Trichuris (T.) trichuria* (whipworm) were treated with mebendazole 200 mg once daily for 3 days. In 1996 this treatment was given after delivery, in 2007 at the time of diagnosis or after the first trimester.

### Malaria data analysis

In this area the natural immunity to malaria is weak because the transmission is very low, so that most patients with malaria parasites become symptomatic. However because of the systematic weekly screening regardless of symptoms, many episodes are detected before symptoms arise. For this reason we cannot strictly speak of incidence or prevalence during the entire follow up period. Furthermore the number of infections of malaria relates not just to transmission but also to the poor response to antimalarial treatment. At the time of the 1996 survey quinine had an estimated failure rate of 23% and mefloquine 28% [58]. This makes it difficult to assign each episode as a new case, as it might be a treatment failure.

In the analysis, women were categorized as “free of malaria” if all the malaria smears done at antenatal visits up to the day of the stool test were negative; women with a positive malaria smear up to the day of the stool test were categorized into one of 3 groups: “*P. vivax* group” or “ *P. falciparum* group” or “ mixed infection group”. Women in the *P.vivax* group only had one or more episodes of *P.vivax*, women in the *P. falciparum* group only had one or more episodes of *P. falciparum* and women in the mixed group may have had a single mixed infection of *P.falciparum* and *P.vivax* or on separate occasions a *P.falciparum* and a *P.vivax*. Results are given as proportions.

### Ethics statement

Following the ANC system as well as participation in the stool survey was voluntary. Providing a stool sample did not involve any risk for the pregnant woman. For these reasons no informed consent was obtained. Pregnancy records have been routinely entered to a data recording system since 1987. Ethical approval for analyzing these patient records was given by the Oxford Tropical Research Ethics Committee (reference: OXTREC 28–09).

### Laboratory

Stool samples were prepared using the formalin–ethyl acetate sedimentation technique [59] and hookworm ova counts performed. Two wet preparations were done for each sample to increase the sensitivity of detection and verify negative slides. The stool assessment was quantitative: a standard dilution of the stool sample was made and 100 µl (taken with a Gilson pipette) was put on a slide. The entire area under the cover slip (22×22 mm) was examined with the X10 objective. Hookworm ova were counted and the number multiplied by 10 was the estimated number of ova per ml of faeces. Other geohelminths were reported as: 1 ova per slide rare, 2–3 per slide 1+, 4–10 per slide 2+ and >10 per slide 3+. In 1996, all stool samples were quality controlled by laboratory staff (WB) from the Liverpool School of Tropical Medicine and Hygiene with good agreement.

Thick and thin malaria smears were stained with Giemsa and examined under oil immersion; the presence of any asexual blood stage parasite was declared as malaria positive. Smears were declared negative after reading 200 fields.

Blood samples (finger prick) were centrifuged at 12000 rpm for 3 minutes and read using a standard haematocrit reader. The haematocrit value measured the day of the stool test was used to describe anaemia. If not available, the result the closest to the stool test day (but within 8 weeks prior to) was chosen.

### Statistical analysis

Data were entered using Microsoft Access, and analyzed using SPSS version 14 for Windows (SPSS, Benelux inc., Gorinchem, Netherlands) and Epi Info (Centre for Disease Control and Prevention). Student's t-test and Mann-Whitney test were used for comparison of means and ranks respectively. Categorical data were compared using the chi-squared test or the Fisher's exact test, as appropriate. To assess independent predictors of malaria, anaemia and LBW, a multivariate unconditional logistic regression model was fitted using the variables that were significantly associated in univariate analysis.

## Results

A total of 829 pregnant women provided a stool sample for examination; this represented 85% (339/401) of the ANC attendees in 1996 and 33% (490/1,485) in 2007. There was no significant difference in the baseline demographics between women who provided a stool sample and those who did not (data not shown).

In 1996 all women were from Maela refugee camp; in 2007, 42% (244/490) were from the camp, while the others attended the migrant antenatal clinics. Participants in the 2007 survey were older (+1.6 years), had their stool test at a later gestational age (+4 wks) and were less anaemic than those in the 1996 survey (Table 1).

**Table 1:** Baseline characteristics of pregnant women enrolled in the 1996 and 2007 surveys.

		1996	2007	P
Total, N (%)		339 (100)	490 (100)	
Age^a^ (in years)		25±6 [15–44]	27±7 [14–46]	**0.001**
Age (in years)				**0.003**
	15–19	67 (19.8)	77 (15.8)	
	20–24	98 (28.9)	140 (28.7)	
	25–29	89 (26.3)	104 (21.3)	
	30–34	54 (15.9)	77 (15.8)	
	35–40+	31 (9.1)	90 (18.4)	
Gravida group				0.133
	G1	72 (21.2)	132 (27.1)	
	G2	73 (21.5)	103 (21.1)	
	G3	51 (15.0)	65 (13.3)	
	G4	56 (16.5)	56 (11.5)	
	G5+	87 (25.7)	132 (27.0)	
EGA at stool test^a^, (weeks)		23±9 [2–40]	27±7 [9–44]	**<0.001**
Hct at stool test^a^		31.5±3.6 [19.5–44.0]	32.3±3.4 [19.0–49.0]	**0.001**
Non-severe anaemia		91 (27)	90 (18)	**0.005**
Severe anaemia		1 (0.3)	1 (0.2)	1.000
Malaria during pregnancy b	Total	90 (27)	96 (20)	**0.022**
	*Pv*	48 (14)	13 (3)	**<0.001**
	*Pv*	36 (11)	65 (13)	**0.036**
	*Pf* & *Pv*	6 (2)	18 (4)	0.204
Nr of episodes^c^		1 [1–4]	2 [1–8]	**<0.001**
Nr intermittent screensc,d		25 [2–39]	25 [2–36]	**0.806**

Data are presented in number, (%), unless stated differently.

Significant results are presented in bold.

EGA = Estimated Gestational Age, Hct = Haematocrit, Pf = *P. falciparum,* Pv = *P. vivax*

a Data presented as mean ± standard deviation, [range].

b At least one smear positive for malaria during intermittent screening during the whole pregnancy.

c Data are median [range].

d Intermittent screening – number of weeks the woman was screened for malaria during the whole pregnancy.

Between 1996 and 2007 there was a significant decline in the proportion of women who had: any malaria, 27% (90/339) *vs.* 20% (96/490), (P = 0.02), *P. falciparum* malaria, 14% (48/339) *vs.* 3% (13/490), (P<0.001) and anaemia, 27% (91/339) *vs.* 18% (90/490), (P = 0.005), but an increase in the proportion of women infected with *P.vivax* (Table 1).

### Proportion and intensity of geohelminth infections

Overall 70% (578) of the 829 women were infected with at least one geohelminth, including hookworm (43% (355)), *A. lumbricoides* (34% (285)) or *T. trichuria* (31% (250)) alone or in combination (Table 2). Prevalence was significantly higher in 1996 than 2007: 81% (95% CI: 76–84) (273/339) *vs.* 62% (95% CI: 58–66) (305/490), P<0.001. The intensity of worm infections was low, with high hookworm ova counts (≥1000 ova/mL) found in <10% of the positive results, and a maximum count of 2900 ova/mL. Hookworm (Table 2) and *T.trichuria* intensities of infection decreased between the 2 surveys, while *A. lumbricoides* decreased in all intensities except the highest group.

**Table 2:** Proportions of soil transmitted geohelminths and hookworm infection intensity in pregnant women by year of survey.

Women infected with geohelminths: year, N		1996, N = 273	2007, N = 305	P
Hookworm only		63 (23)	78 (26)	0.485
Hookworm and *T.trichuris*		43 (16)	38 (12)	0.255
Hookworm and *A. lumbricoides*		41 (15)	30 (10)	0.058
*A. lumbricoides*, hookworm and *T.trichuria*		41 (15)	21 (7)	**0.012**
*A. lumbricoides* only		46 (17)	60 (20)	0.382
*A. lumbricoides* and *T.trichuris*		20 (7)	26 (8)	0.595
*T.trichuria* only		19 (7)	52 (17)	**<0.001**
More than 1 geohelminth		145 (53)	115 (38)	0.079
Any hookworm		188 (69)	167 (55)	**<0.001**
Intensity of HW infection (ova/mL faeces)	1–499	110/188 (59)	137/167 (82)	
	500–999	51/188 (27)	21/167 (13)	
	≥1,000	27/188 (14)	9/167 (5)	

Data are presented in number, (%). Significant results are presented in bold.

### Geohelminths and malaria

Thirty three pregnant women had their first malaria infection after stool testing and were excluded from further analysis related to geohelminths and malaria and anaemia.

For the purpose of this analysis geohelminth infections were assumed to be present until mebendazole treatment was administered, as there was no routine deworming policy.

Overall 153/796 women (19%) had malaria detected at least once prior to, or at the day of the stool test. Most of the women presented with single species infections; 35% (53) had *P. falciparum* infections only and 54% (83) *P. vivax* only. *P. falciparum* and *P. vivax* simultaneously or on separate occasions occurred in the remaining 11% (17).

**Figure 1:**
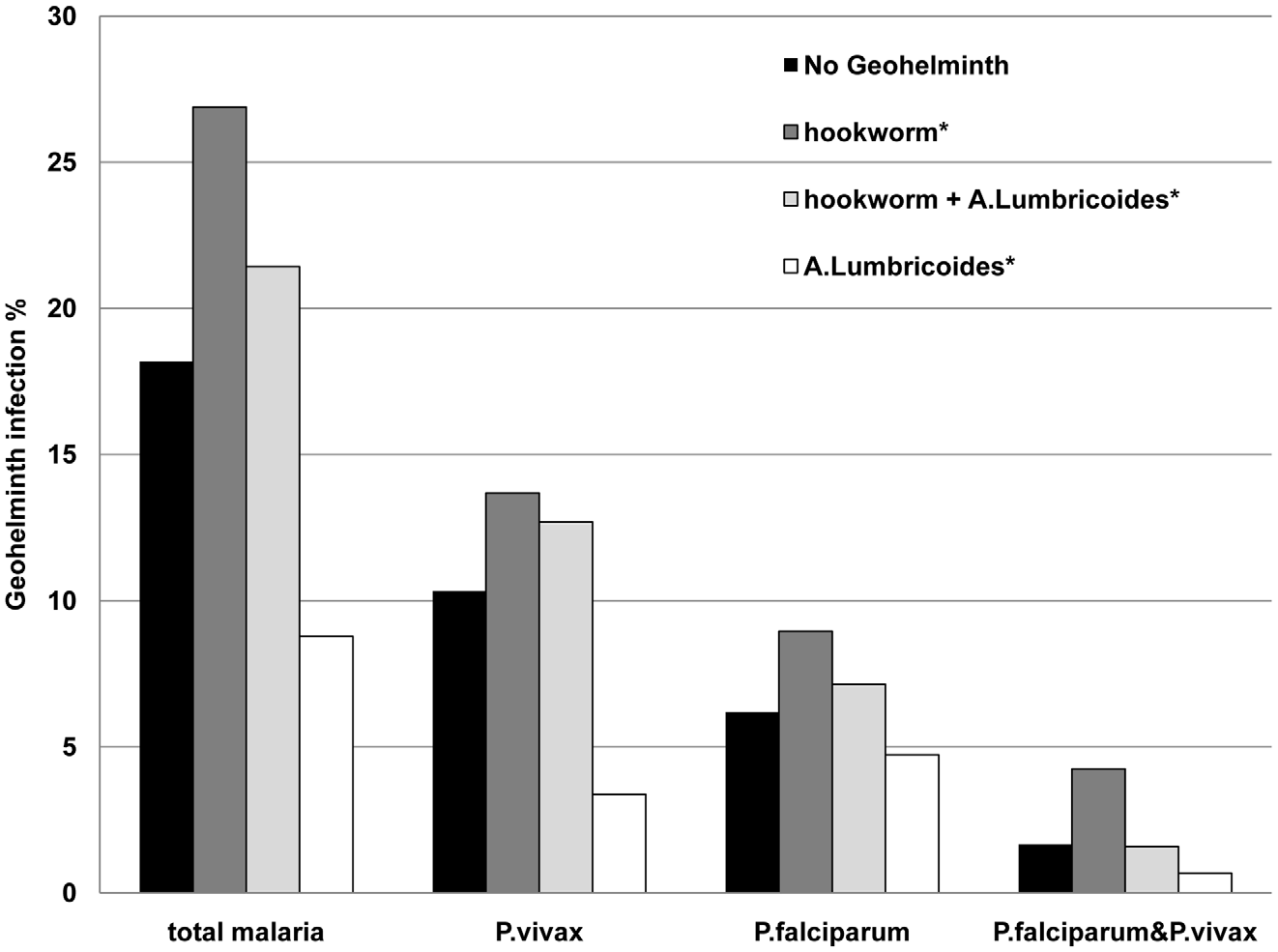
The proportion of women with geohelminth infection by malaria species. Footnote: *  =  +/- *Trichuris trichuria*.

**Figure 2:**
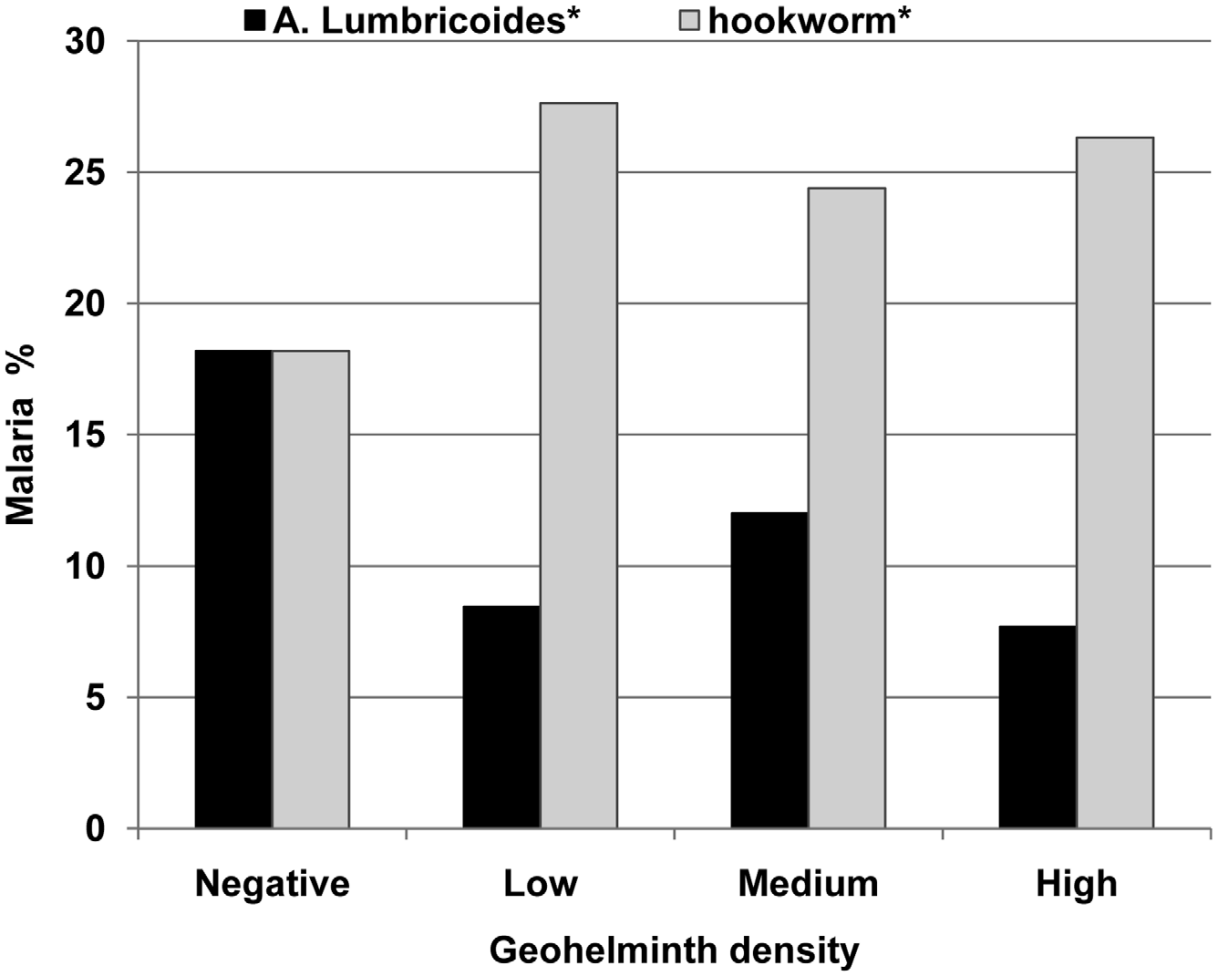
The proportion of women with malaria by density of geohelminth. Footnote: Low, medium and high defined as >0–499, 500–999, ≥1000 ova/ml for hookworm and 1–3, 4–10, >10 ova per slide for *A. lumbricoides*, respectively. *  =  +/− *Trichuris trichuria*.

The proportion of women with malaria in pregnancy was similar in those with geohelminth co-infection or without: 18% (44/242) *vs.* 20% (109/554), P = 0.77 (Table 3). There were important differences in the proportions of women with malaria depending on the type of geohelminth co-infection (Figure 1). The highest proportions of both *P. falciparum* and *P.vivax* malaria were seen with hookworm (±*T. trichuria*) co-infections and the lowest with *A. lumbricoides* (±*T. trichuria*) co-infections. The protective effect of *A. lumbricoides* (±*T. trichuria*) remained significant for *P.vivax* malaria when stratifying by malaria species (Table 3). The overall proportion of women with malaria in women with *A. lumbricoides* infections was approximately half that in hookworm infections. Temperature, days of fever, number of episodes of malaria and parasitaemia were not significantly different between the worm groups (data not shown). There were only 3 women with hyper-parasitaemic malaria (more than 4% of the red blood cells infected with *P. falciparum*), 2 of them without worm infection, and 1 woman with hookworm infection. The relationship between malaria and stool ova counts for the hookworm (±*T. trichuria*) group and for ova count in the *A. lumbricoides* (±*T. richuria*) group was explored (Figure 2). There was no relationship between malaria and the hookworm (P = 0.76) or *A. lumbricoides* (P = 0.92) stool ova counts. There was not sufficient data to study interaction between geohelminth single infections and their association with malaria, as nearly half of all infections were combinations of worms (Table 2).

**Table 3:** Risk of malaria by geohelminth group.

	stool result	*Pf* only (%)	AOR (95% CI)	*Pv* only (%)	AOR (95% CI)	Total malaria (%)	AOR (95% CI)
**geohelminth**	+	38/483 (7.9)	0.89 (0.48–1.65)	58/503 (11.5)	0.97 (0.59–1.59)	109/554 (19.7)	1.10 (0.75–1.63)
	−	15/213 (7.0)	1.00	25/223 (11.2)	1.00	44/242 (18.2)	1.00
**HW** *	+	19/174 (10.9)	1.62 (0.80–3.29)	29/184 (15.8)	1.48 (0.83–2.63)	57/212 (26.9)	**1.66 (1.06–2.59)**
	−	15/213 (7.0)	1.00	25/223 (11.2)	1.00	44/242 (18.2)	1.00
**HW&AL** *	+	9/108 (8.3)	1.20 (0.51–2.84)	16/115 (13.9)	1.28 (0.65–2.51)	27/126 (21.4)	1.23 (0.72–2.10)
	−	15/213 (7.0)	1.00	25/223 (11.2)	1.00	44/242 (18.2)	1.00
***AL*** *	+	7/142 (4.9)	0.68 (0.27–1.72)	5/140 (3.6)	**0.29 (0.11–0.79)**	13/148 (8.8)	**0.43 (0.23–0.84)**
	−	15/213 (7.0)	1.00	25/223 (11.2)	1.00	44/242 (18.2)	1.00

* =  +/− Trichuris trichuria.

*PF* = *P. falciparum, PV* = *P. vivax, AOR* = Adjusted Odds Ratio, 95% CI = 95% Confidence Interval, HW = Hookworm, AL = *A. lumbricoides* Significant results are presented in bold.

**Figure 3:**
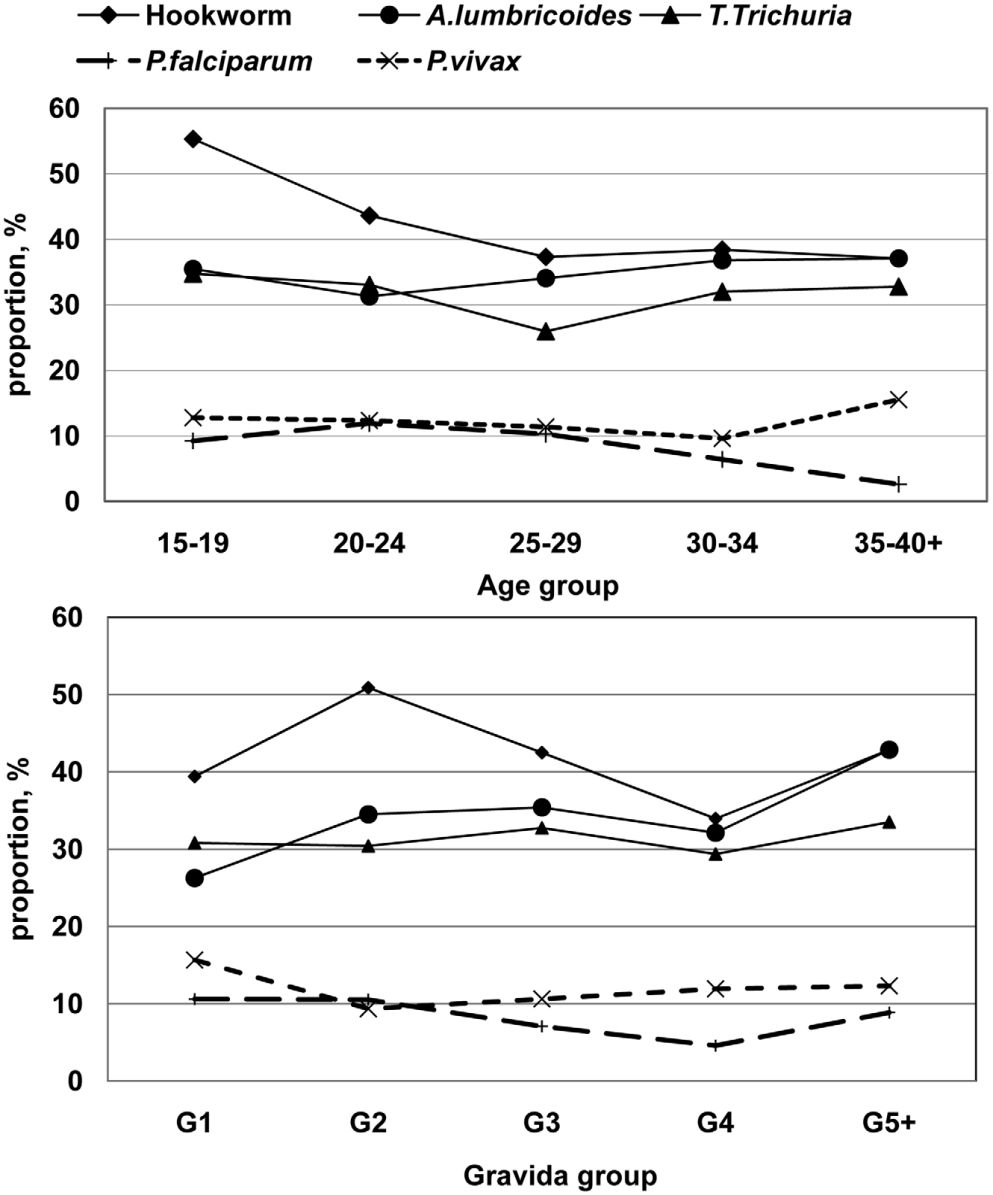
Proportion of geohelminths and malaria by age and gravid group among 794 pregnant women.

The proportions, by age and gravid groups, of women with malaria and those with geohelminths are presented (Figure 3). Age was not significantly associated with malaria, but gravidity was: 25% (49/198) in primigravida *vs.* 17% (104/595) in multigravida, P = 0.029. The proportion of hookworm infection was higher in teenage women, although this was not significant.

In a multiple regression model, primigravida (AOR: 1.53, 95% CI: 1.01–2.32, P = 0.043) and hookworm co-infection (AOR: 1.66, 95% CI: 1.06–2.60, P = 0.027) remained the two independent factors associated with an increased risk of malaria while the protective effect of *A. lumbricoides* co-infection remained significant (AOR: 0.44, 95% CI: 0.23–0.86, P = 0.015). Year of survey and ova counts for hookworm and *A. lumbricoides* were non-significant.

### Geohelminths and anaemia

Sixty five women (8%) did not have a haematocrit measurement at the time of or before the stool test and were not included in this part of the analysis. Mean haematocrit was similar whether geohelminth infection was present or not in the remaining 733 pregnant women. The proportion of women with anaemia was higher in women with high intensity hookworm infection compared to those with lower counts, 41% (14/34) *vs.* 21% (150/699), (P = 0.011); in multigravida compared with primigravida, 24% (134/547) *vs*. 16% (30/183), (P = 0.024); those who were older than 25 years, 25% (100/394) *vs.* 19% (64/337), (P = 0.041); and those who had malaria, 30% (45/152) *vs.* 20% (119/581), (P = 0.021). In a logistic regression model high hookworm load (AOR: 2.05, 95% CI: 1.01–4.20), P = 0.049), malaria (AOR: 1.83, 95% CI: 1.12–2.74, P = 0.004), being multigravid (AOR: 1.79, 95% CI: 1.15–2.78, P = 0.009) and participating in the 1996 survey (AOR: 1.57, 95% CI: 1.03–2.08, P = 0.032) remained independently associated with anaemia.

### Geohelminths, pregnancy outcomes and risk for low birth weight

Pregnancy outcome data were available for 94% (783/829) of women. There were 14 abortions (2%) and 8 stillbirths (1%). Eleven infants were born with congenital abnormalities (8 live-births and 3 stillbirths). Neither stillbirth nor congenital abnormalities were significantly associated with geohelminth infection. The mean gestational age at delivery was 38.9±1.7 [28.4–42.5] weeks. Mean gestational age and the proportion of premature infants were not significantly different in the presence or absence of geohelminth infection.

Birth weight data were available for 87% (648/748) of live-born, normal, singletons. Mean birth weight was 2900±447 [1100–4400] g. The proportion of LBW newborns was significantly higher among primigravida compared to multigravida (25% (41/162) *vs.* 10% (46/483), P<0.001), in women aged <25 years *vs.* older (19% (57/301) *vs.* 9% (30/345), P<0.001), with hookworm infection *vs.* none (17% (46/278) *vs.* 11% (41/370), P = 0.048), and in premature *vs.* term infants (58% (34/59) *vs*. 9% (53/589), P<0.001). Anaemia and malaria were not significant risk factors for LBW. In a logistic regression model excluding prematurity (the strongest risk factor for LBW (n = 59)), the presence of hookworm infection was independently but weakly associated with LBW (AOR: 1.81, 95% CI: 1.02–3.23, P = 0.041) as was (more significantly so) being primigravid (AOR: 3.27, 95% CI: 1.83–5.84, P<0.001).

## Discussion

The proportion of intestinal geohelminth infections in pregnant women on the Thai-Burmese border is high and comparable to reports from other parts of South East Asia, including Thailand, Burma and Vietnam [60]–[62], while malaria transmission is low [50]. The cross-sectional surveys conducted 11 years apart confirmed a declining prevalence of intestinal parasites but prevalence remains higher than the 20–30% WHO criteria for mass deworming [3], [63]. Anaemia in this population is common but predominantly mild [64] and the association of hookworm infection and anaemia was only significant for the highest intensity of hookworm infection, a finding already reported half a century ago [65].

This site has a unique system of antenatal care established in 1986 in response to a high malaria related-maternal mortality rate(1000/100,000 live births)[52] and lack of any drug to offer as chemoprophylaxis due to multidrug resistant strains of *P.falciparum*. Women are encouraged to attend ANC on a weekly basis. Attendance is high and most women average more than 10 consultations per pregnancy in both the refugee and migrant settings. Women with detectable parasitaemia on screening are treated regardless of symptoms as they are unlikely to clear parasitaemia without becoming symptomatic [66]. The reduction in malaria (and anaemia) incidence in pregnancy in this population has been described in detail elsewhere [67].

This is the first time, to our knowledge, that *A. lumbricoides* infection has been associated with a reduced risk of *P. vivax.* The novelty of this finding might be influenced by the fact that many studies on interactions between worms and malaria were done in Africa, where *P. falciparum* is the predominant species. Other investigators in African settings have described higher prevalence of falciparum malaria in presence of *A. lumbricoides* in pregnant women [49] and in children [29]. These differences could be related to acquired immunity, methodological issues or to interactions (other helminths could alter the immune response e.g. schistomiasis or *Strongyloides stercoralis*). The density of infection of hookworm and *A. lumbricoides* in these surveys was low, as is usually reported from Asian settings, and no relationship was found between malaria and geohelminth density.

Similar to a study in Kenyan pregnant women *A. lumbricoides* prevalence increased with gravidity, but whereas they observed the same trend with maternal age this was not observed on the Thai-Burmese border[48]. Hookworm prevalence peaked amongst the lowest age group, and reached a plateau after 25 years of age, which is similar to the pattern reported from Kenyan pregnant women[48].

Co-infection with hookworm was associated in our setting with a significantly higher risk of malaria, but this did not reach statistical significance for the individual plasmodial species. Similar associations have been previously reported for *P. falciparum* in children [68], [69], adults [19] and pregnant women [48]. In regions of high prevalence it is plausible that helminthes might suppress the ability to clear infections (resulting in a positive association between helminth infection and asymptomatic malaria parasitaemia), or suppress the inflammatory responses that result in clinical disease (resulting in a negative association between helminth infection and clinical malaria disease) [21] but this seems unlikely in this setting where malaria prevalence is low. Subclinical haematological cues may influence the host attractiveness for the vector [19]. In Thailand hookworm was shown to be associated with increased incidence of *P. falciparum* but not *P.vivax* malaria [19]. In our setting, the replenishment of pregnant women's iron and folate reserves may have resulted in reticulocytosis and may have increased *P.vivax* densities [70]. This effect would be expected to be greatest in hookworm infections, and may well explain why the interaction could be observed in our study whereas it could not be in the study of Nacher [19] where patients did not receive haematinics. *P. falciparum* and to a much greater extent *P. vivax* prefer to invade young red cells. This would not account for the negative association seen between malaria and *A. lumbricoides*. However, an alternative hypothesis proposed by some authors is that residential location and spatial aspects of exposure may explain some of the associations between worms and malaria [71]. NGOs in the refugee camps have provided intermittent deworming (6–12 monthly single dose mebendazole) to school children since late 2001. There has been no deworming program for pregnant women or adults. It is likely that sporadic deworming of children and improved footwear or sanitation, has led to the decreased proportion of geohelminths in pregnant women observed between the two survey periods.

Active weekly screening as part of routine antenatal care has made severe malaria a rare event. No association between disease severity and the prevalence of geohelminths could be demonstrated. The decreased proportion of women with mild anaemia between the two surveys could be related to the decrease of geohelminth infection; it also could be due to the reduction in *P. falciparum* observed on the Thai-Burmese border [72], [73] or to the implementation of anaemia prophylaxis for all pregnant women. In Sierra Leone, the administration of iron and folate supplements had a greater effect on haematocrit than the administration of albendazole [11]. This suggests that deworming to prevent anaemia should not be used as sole strategy against anaemia [74]. If *A. lumbricoides* coinfection does indeed attenuate malaria, then mass deworming may reduce a potential protective benefit. On the other hand hookworm was associated with a higher proportion of malaria, low birth weight and anaemia suggesting that hookworm should be treated in pregnancy. As there is no specie selective antihelminth at hand, deworming policies should be based on local prevalence and intensity of geohelminths, malaria, and anaemia severity.

The present paper has limitations: the surveys were cross sectional, pooling data from different periods and the sample size may not have been sufficient to detect quantitative effects of the different worm species on different plasmodial species. No plausible explanation has been provided for the observed associations. No socioeconomic, behavioral or environmental factors were available for analysis, however these tend to be uniformly similar across the population of refugees and migrant workers: These people live in poor conditions and all are economically deprived so that it is unlikely to be a confounder in the analysis. The assumption that worms observed in the stool sample were present at the time of malaria is plausible at the population level given the lifespan of worms but it is not possible to ascertain that was always the case in each individual. This may have reduced the precision. Nevertheless, the present paper presents for the first time in the same data set a range of complex interactions between hookworm, *A. lumbricoides* and both *P. falciparum* and *P. vivax* malaria, during pregnancy. Our findings potentially have considerable practical and evolutionary implications. Future trials to confirm or deny the associations observed here require well designed longitudinal studies to account for the observed complex and conflicting interactions.
